# Supplementary material for: Neutralizing antibodies elicited in macaques recognize V3 residues on altered conformations of HIV-1 Env trimer
Source: NPJ Vaccines. 2024 Dec 5;9:240. doi: 10.1038/s41541-024-01038-0 (PMC11621127; doi:10.1038/s41541-024-01038-0)
Supplement: Supplementary file 1 — Supplementary information [file 41541_2024_1038_MOESM1_ESM.pdf]

Supplementary Figures

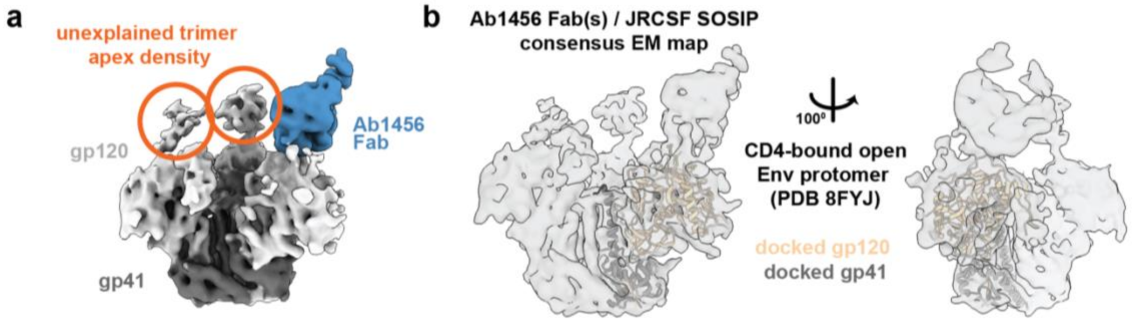

Supplementary Figure 1: Analysis of Ab1456 Fab(s) / JRCSF SOSIP consensus map. a, Unexplained density present at the trimer apex of the consensus EM map. **b**, Consensus EM density of the Ab1456 / JRCSF structure including a cartoon representation of docked coordinates of protomer A gp120 and gp41 from an Env heterotrimer (HT2) bound by two copies of soluble CD4 (PDB 8FYJ).

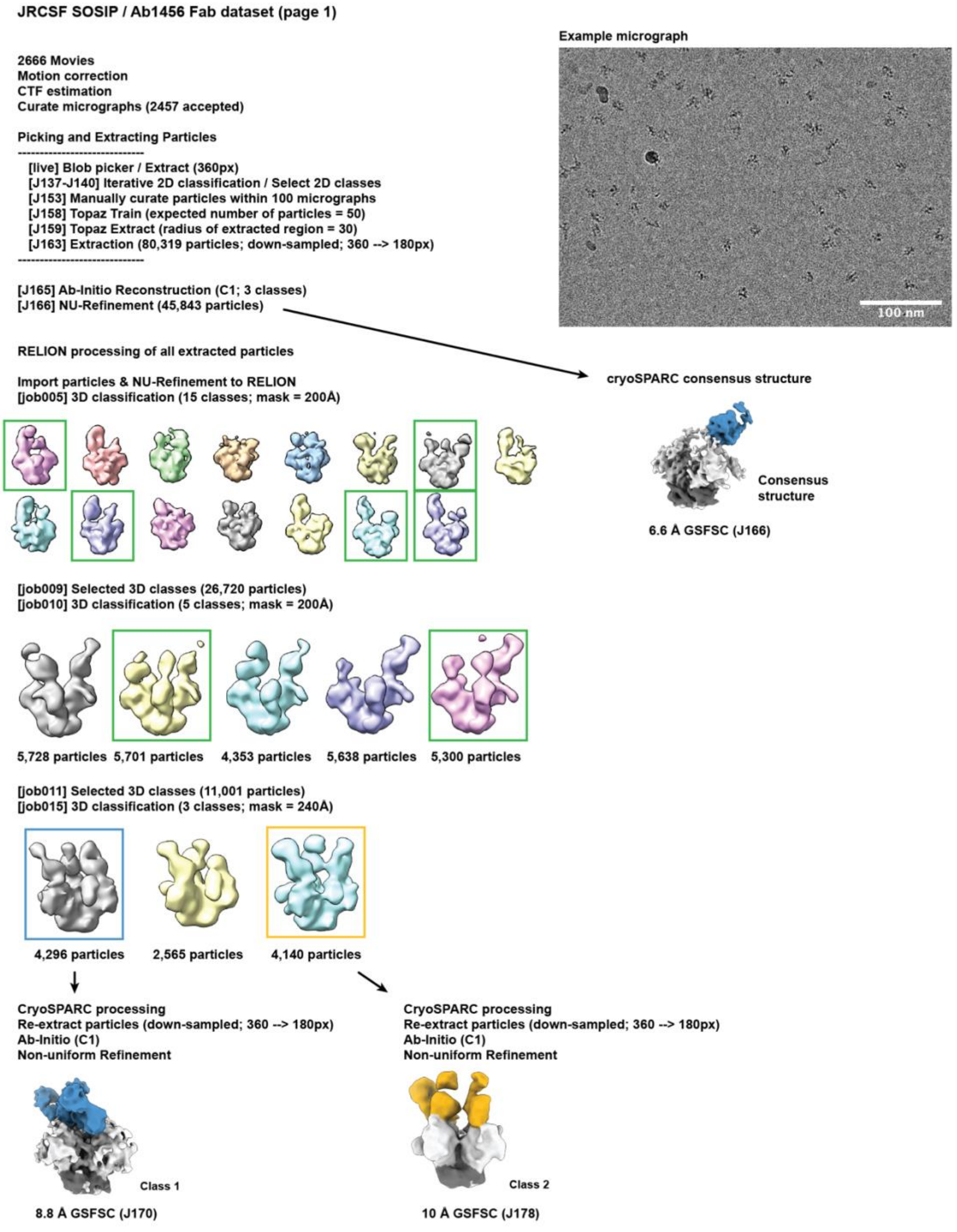

Supplementary Figure 2 (page 1 of 3): Data processing of the Ab1456 Fab / JRCSF SOSIP dataset.

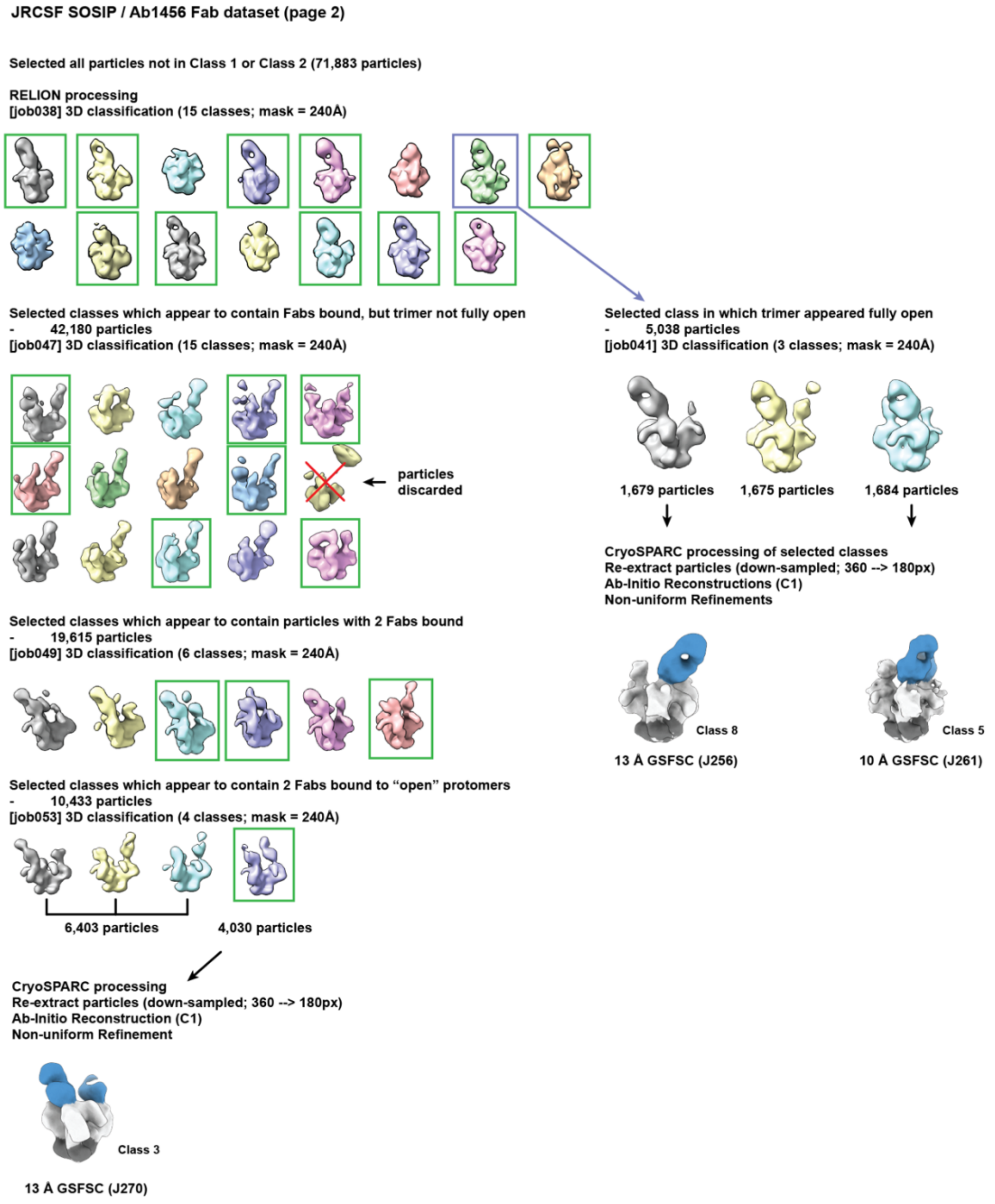

Supplementary Figure 2 (page 2 of 3): Data processing of the Ab1456 Fab / JRCSF SOSIP dataset.

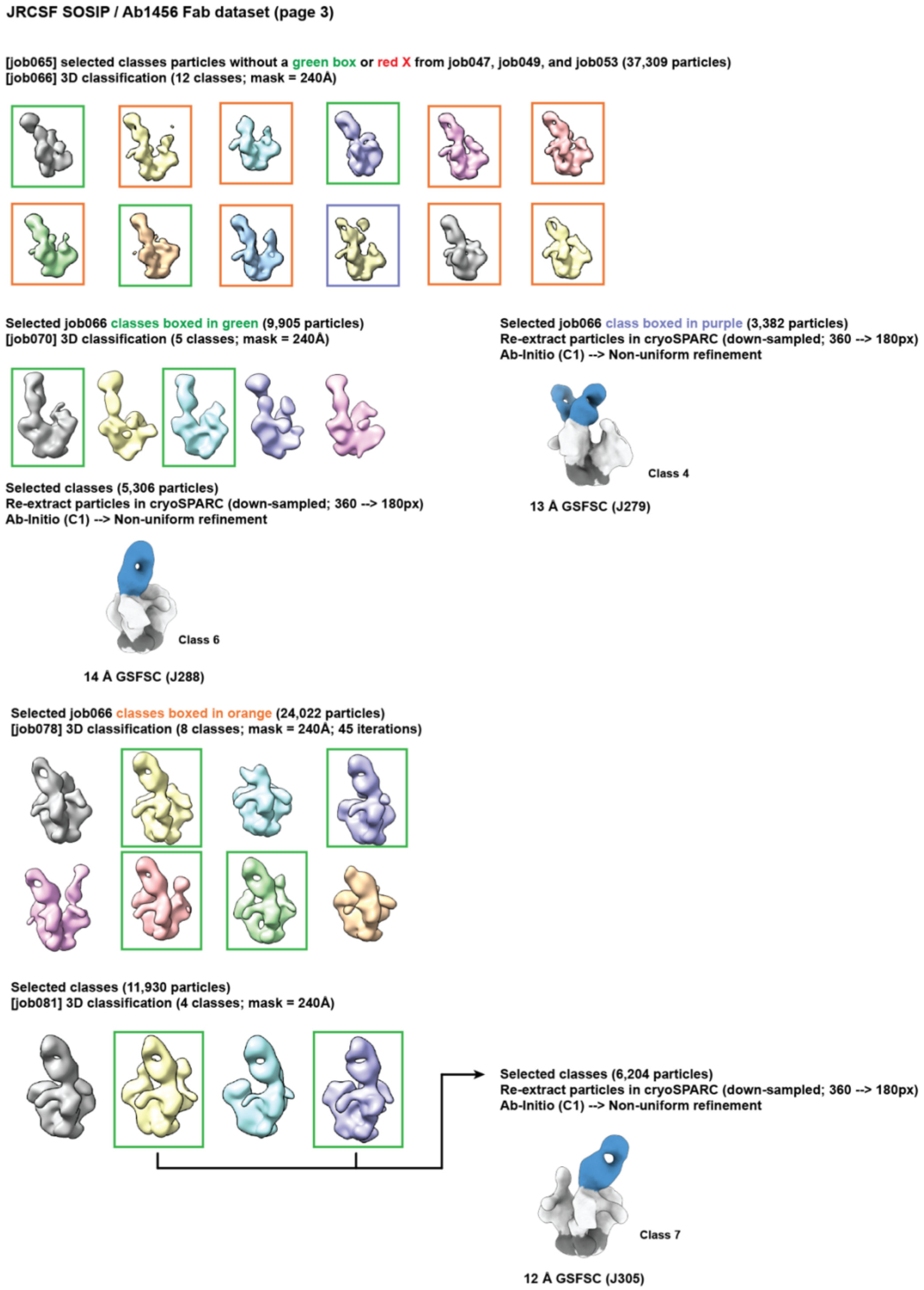

Supplementary Figure 2 (page 3 of 3): Data processing of the Ab1456 Fab / JRCSF SOSIP dataset.

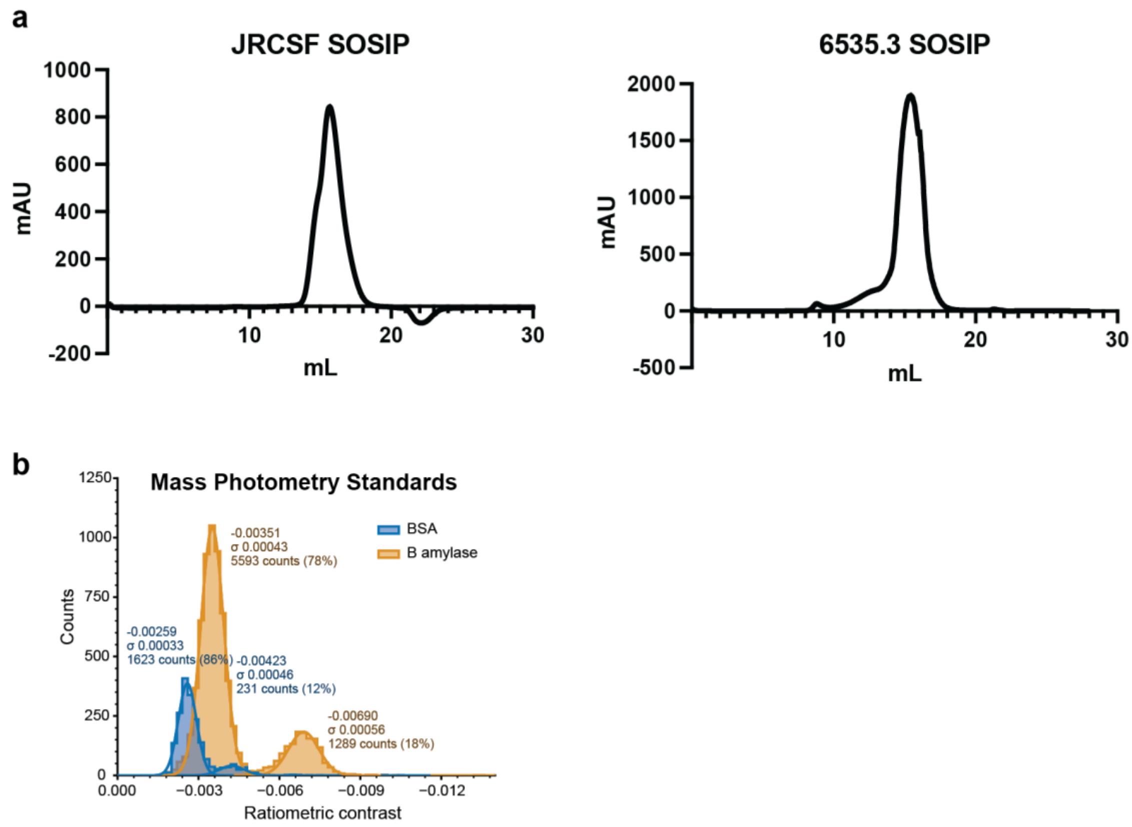

Supplementary Figure 3: Characterization of JRCSF and 6535.3 SOSIP Envs. a, SEC profiles for the purification of JRCSF SOSIP (left) and 6535.3 SOSIP (right). **b**, Experimental histograms for the mass standards used in mass photometry experiments of SOSIP Envs and Fab-SOSIP complexes.

6535.3 SOSIP / Ab1271 Fab dataset

Picking and Extracting Particles

- [J50] Manually picked particles within 25 micrographs
- [J53] Topaz Train (expected number of particles = 75)
- [J58] Topaz Extract (from 201 micrographs)
- [J61] Manually curate particles within 201 micrographs
- [J63] Topaz Train (expected number of particles = 100)
- [J66] Topaz Extract
- [J69] Extraction (down-sampled; 300 --> 150 px)
- [J70/J71] 2D classification / Select 2D classes
- [J112] Extract from Micrographs (360 px) -- 134,369 particles

----

[J117] Ab-Initio (C1)
[J118] Homogeneous refinement (C1)

Example micrograph

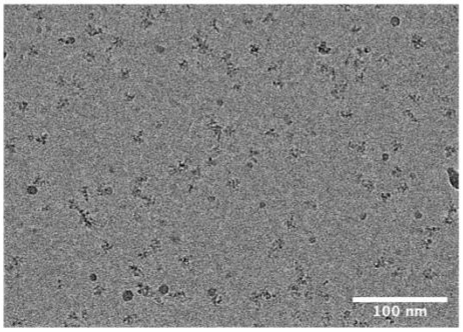

RELION processing

Import particles & consensus refinement
3D classification (6 classes)

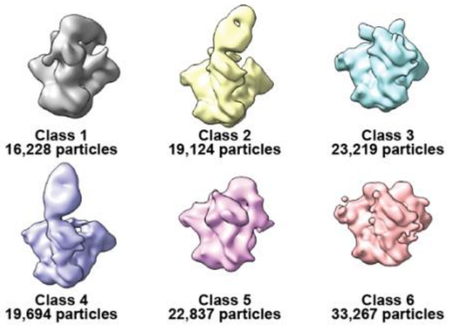

CryoSPARC processing of unbound trimer
Re-extract particles from RELION Class 3 + Class 6
Ab-Initio (C1)

Non-uniform Refinement

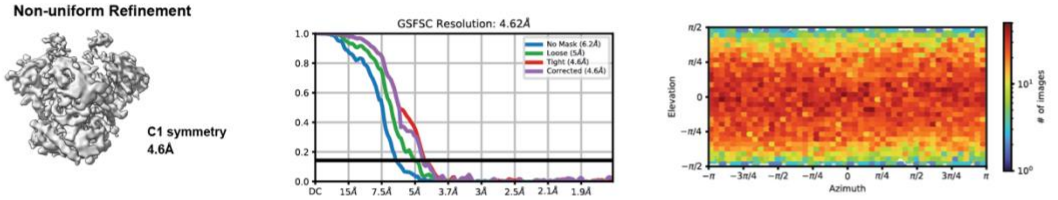

CryoSPARC processing of Ab1271 Fab-bound trimer
Re-extract particles from RELION Class 2 + Class 4
Ab-Initio (C1)

Non-uniform Refinement

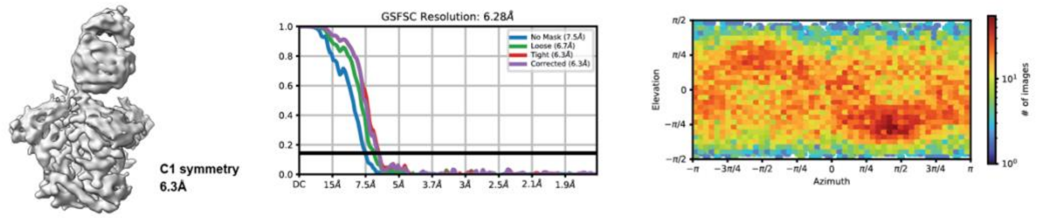

Supplementary Figure 4: Data processing of the Ab1271 Fab / 6535.3 SOSIP dataset.

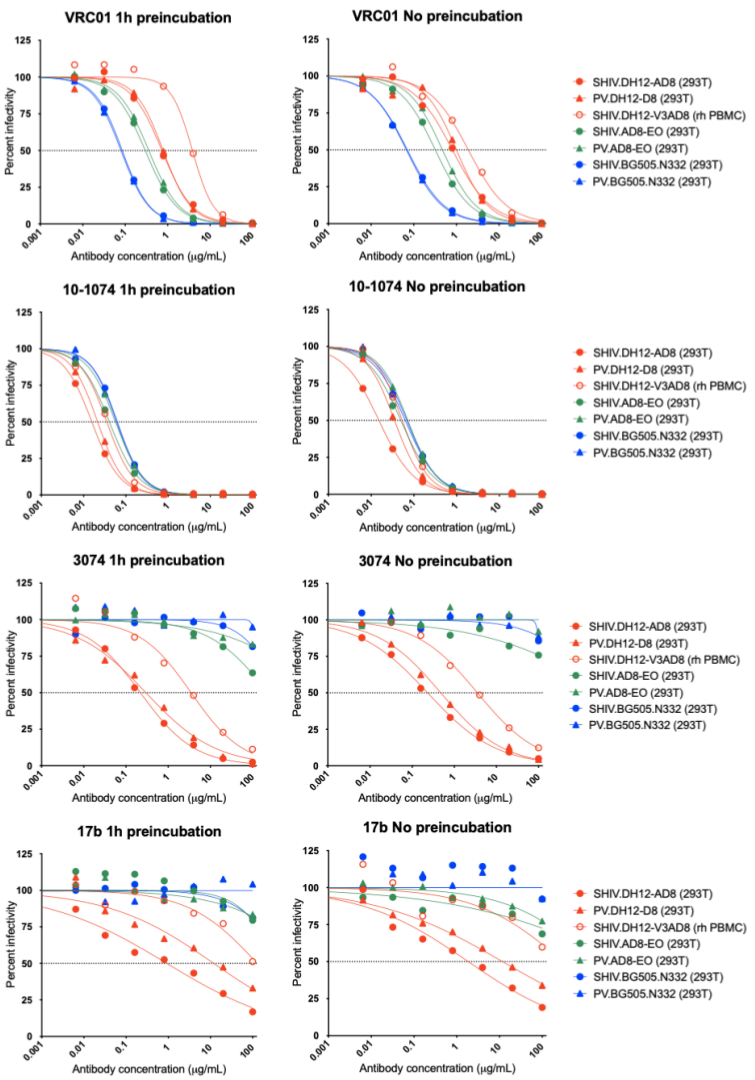

	VRC01	
	1 hour preincubation	No preincubation
SHIV.DH12-V3AD8 (293T)	0.734	0.806
PV.DH12-V3AD8 (293T)	0.784	1.00
SHIV.DH12-V3AD8 (rh PBMC)	3.85	1.90
SHIV.AD8-EO (293T)	0.297	0.320
PV.AD8-EO (293T)	0.360	0.471
SHIV.BG505.N332 (293T)	0.085	0.071
PV.BG505.N332 (293T)	0.080	0.067

	10-1074	
	1 hour preincubation	No preincubation
SHIV.DH12-V3AD8 (293T)	0.016	0.015
PV.DH12-V3AD8 (293T)	0.021	0.035
SHIV.DH12-V3AD8 (rh PBMC)	0.035	0.054
SHIV.AD8-EO (293T)	0.041	0.050
PV.AD8-EO (293T)	0.057	0.072
SHIV.BG505.N332 (293T)	0.063	0.063
PV.BG505.N332 (293T)	0.060	0.069

	3074	
	1 hour preincubation	No preincubation
SHIV.DH12-V3AD8 (293T)	0.220	0.233
PV.DH12-V3AD8 (293T)	0.297	0.477
SHIV.DH12-V3AD8 (rh PBMC)	3.50	3.54
SHIV.AD8-EO (293T)	>100	>100
PV.AD8-EO (293T)	>100	>100
SHIV.BG505.N332 (293T)	>100	>100
PV.BG505.N332 (293T)	>100	>100

	17b	
	1 hour preincubation	No preincubation
SHIV.DH12-V3AD8 (293T)	0.918	1.86
PV.DH12-V3AD8 (293T)	11.9	11.8
SHIV.DH12-V3AD8 (rh PBMC)	>100	>100
SHIV.AD8-EO (293T)	>100	>100
PV.AD8-EO (293T)	>100	>100
SHIV.BG505.N332 (293T)	>100	>100
PV.BG505.N332 (293T)	>100	>100

Supplementary Figure 5: Omission of the antibody-virus preincubation step does not affect the potency of bNAbs or antibodies recognizing non-closed Env trimers. The sensitivity of viruses expressing the DH12-V3AD8 (red), AD8-EO (green) and BG505.N332 (blue) Envs to neutralization by VRC01, 10-1074, 3074, and 17b in a standard TZMbl assay including a 1 hour antibody and virus preincubation step[41,42] and a modified assay with no preincubation are shown. Neutralization curves for the indicated mAbs are shown on the left (dashed lines indicate 50% reduction in virus infectivity), with the corresponding 50% inhibitory concentrations (IC_{50}) in µg/mL shown on the right (coloring indicates relative neutralization potency). Pseudoviruses (PV) as well as replication-competent SHIVs derived either by HEK293T transfection (293T) or following propagation in rhesus macaque PBMC (rh PBMC) were tested. Note that similar to Ab1456 (Fig. 4a), the slopes of the neutralization curves for the linear V3 mAb 3074 and the CD4-induced mAb 17b are more shallow slopes than slopes of the CD4bs bNAb VRC01[46] and the V3 glycan bNAb 10-1074.[45]

	Ab1456 Fab / JRCSF SOSIP	Ab1271 Fab / 6535.3 SOSIP
**Data collection conditions**		
Microscope	Titan Krios	Titan Krios
Voltage (kV)	300	300
Camera	Gatan K3 6k x 4k	Gatan K3 6k x 4k
Energy filter slit width (eV)	10	10
Magnification	105,000x	105,000x
Frames per movie	40	40
Recording mode	counting	counting
Dose rate (e^-/pixel/s)	25	26
Total electron dose (e^-/Å^2)	60	60
Defocus range (μm)	-1 to -3	-1 to -3
Pixel size (Å)	0.416 (super resolution)	0.416 (super resolution)
Micrographs collected	2664	2898
Micrographs used	2456	2756
Total extracted particles	80,319	134,369

	Consensus	Class 1	Class 2	Class 3	Class 4	Class 5	Class 6	Class 7	Class 8	Unbound 6535.3 SOSIP	Ab1271 Fab-bound 6535.3 SOSIP
**EMD**	**45944**	**45945**	**45946**	**45947**	**45948**	**45949**	**45950**	**45951**	**45952**	**45942**	**45943**
Particles in class	45,843	4296	4140	4030	3382	1684	5306	6204	1679	56,486	38,818
Symmetry	C1	C1	C1	C1	C1	C1	C1	C1	C1	C1	C1
Map resolution (Å)	6.6	8.8	10	13	13	10	14	12	13	4.6	6.3
FSC Threshold	0.143	0.143	0.143	0.143	0.143	0.143	0.143	0.143	0.143	0.143	0.143

Supplementary Table 1: EM data collection and processing statistics.
